# The Seagrass Effect Turned Upside Down Changes the Prospective of Sea Urchin Survival and Landscape Implications

**DOI:** 10.1371/journal.pone.0164294

**Published:** 2016-10-26

**Authors:** Simone Farina, Ivan Guala, Silvia Oliva, Luigi Piazzi, Rodrigo Pires da Silva, Giulia Ceccherelli

**Affiliations:** 1 IMC - International Marine Centre, Loc. Sa Mardini Torregrande, 09170, Oristano, Italy; 2 Department of Science for Nature and Environmental Resources – University of Sassari, Via Piandanna 4, 07100, Sassari, Italy; University of Connecticut, UNITED STATES

## Abstract

Habitat structure plays an important mediating role in predator-prey interactions. However the effects are strongly dependent on regional predator pools, which can drive predation risk in habitats with very similar structure in opposite directions. In the Mediterranean Sea predation on juvenile sea urchins is commonly known to be regulated by seagrass structure. In this study we test whether the possibility for juvenile *Paracentrotus lividus* to be predated changes in relation to the fragmentation of the seagrass *Posidonia oceanica* (four habitat classes: continuous, low-fragmentation, high-fragmentation and rocks), and to the spatial arrangement of such habitat classes at a landscape scale. Sea urchin predation risk was measured in a 20-day field experiment on tethered individuals placed in three square areas 35×35 m^2^ in size. Variability of both landscape and habitat structural attributes was assessed at the sampling grain 5×5 m^2^. Predation risk changed among landscapes, as it was lower where more ‘rocks’, and thus less seagrass, were present. The higher risk was found in the ‘continuous’ *P*. *oceanica* rather than in the low-fragmentation, high-fragmentation and rock habitats (p-values = 0.0149, 0.00008, and 0.0001, respectively). Therefore, the expectation that juvenile *P*. *lividus* survival would have been higher in the ‘continuous’ seagrass habitat, which would have served as shelter from high fish predation pressure, was not met. Predation risk changed across habitats due to different success between attack types: benthic attacks (mostly from whelks) were overall much more effective than those due to fish activity, the former type being associated with the ‘continuous’ seagrass habitat. Fish predation on juvenile sea urchins on rocks and ‘high-fragmentation’ habitat was less likely than benthic predation in the ‘continuous’ seagrass, with the low seagrass patch complexity increasing benthic activity. Future research should be aimed at investigating, derived from the complex indirect interactions among species, how top-down control in marine reserves can modify seagrass habitat effects.

## Introduction

Predation is a key selective force acting on the morphological, life history and behavioral traits of prey [1,2]. Traits such as armoring and chemical defenses, crypsis and behavioral avoidance of predation have a major influence on fitness in environments with high levels of predation [1,3]. Predation risk, the animal’s likelihood of predator-induced mortality, can strongly influence community dynamics through its effects on prey foraging decisions that often involve habitat shifts [4]. Thus, habitat structure may play an important mediating role in predator-prey interactions either facilitating or hampering both the survival of the prey and the hunting success of the predator. Some habitat characteristics, such as openness, may have opposite effects for the prey, being positive with regard to foraging while negative with regard to predation risk [5–9]. Habitat structure may affect in different ways a recognizable ‘landscape of fear’ for prey species, as animals could alter the use of an area in trying to reduce vulnerability to predation [10]. Effectively, the predation process is considered among the strongest species interactions and, apart from direct effects on the prey’s fitness (resulting in prey death), predation can have a series of non-consumptive effects, altering individual characteristics in prey populations [11].

Predation effectiveness is strongly mediated by the architectural or structural complexity of habitats, which can have contrasting effects [12]: the structure can significantly lower predation risk when it serves as a refuge for prey [13] but can also increase susceptibility to predators that use structure for ambush or camouflage [14,15]. The scenario is even more complicated by the evidence that the effects of habitat structure are strongly dependent on regional predator pools: indeed, they would be largely a function of predator identity, which determines whether habitat complexity either reduces or enhances top-down control within the ecosystem [16,17].

Similar to trees on land, seagrasses are among the main structural agents in marine coastal systems. Seagrass meadows provide a suitable habitat for invertebrate taxa, where leaf canopy plays a central role: it can increase food availability and living space and enhance refuge from predators [18,19] by providing shade [20–22] and baffling currents [23–26].

In the Mediterranean Sea, *Posidonia oceanica* (L.) Delile is the dominant seagrass, forming extensive meadows and structuring habitats of high complexity which provide potential refuges for great variety of species [27–30]. One of them is the sea urchin *Paracentrotus lividus* (Lamarck, 1816), which also commonly occurs in shallow subtidal rocky habitats. *P*. *lividus* is one of the main herbivores in the Mediterranean, playing a central role in the trophic cascade that involves predatory fish, sea urchins and macroalgae as well as seagrasses [31,32]. However, *P*. *lividus* is described as a keystone species on rocky macroalgal communities for the dramatic effects of its high-density populations [33–35].

Complex spatial and habitat-dependent processes shape demographic fate of *P*. *lividus* in Mediterranean. Regional scale factors determine larval availability and settlement patterns of the pelagic stages such as eutrophication (*i*.*e*., promoting larval supply) [36]. Once in the benthos, processes linked to local-scale habitat features become crucial in controlling the population outcome: adult numbers in seagrass meadows are most likely influenced by factors such as local migration (from adjoining rocky habitats) functioning at much smaller scales [37–39], but this is tightly linked to fish predator numbers and refuge availability [40–42].

The main sea urchin predators in the Mediterranean Sea are the fish species *Coris julis*, *Diplodus* spp. and *Sparus aurata* (Linnaeus, 1758): visual hunters of individuals of small and medium sizes across shallow subtidal rocky habitats [43]. However, there are also benthic chemotactic sea urchin predators, such as the *Muricidae* species (*i*.*e*., whelks) [44] and the sea star *Marthasterias glacialis* (Linnaeus, 1758) [45], whose effects in shaping prey populations have been considered negligible, as evidence of their predation has only been occasionally provided [46,47].

Furthermore, little is known about how each prey-predator relationship differs between areas with contrasting landscapes, including heterogeneous landscapes offering a variety of refuges and foraging sites of different quality and homogenous areas consisting of a single habitat type. The consequences of landscape heterogeneity may be particularly important when the presence of predators is highly unpredictable. In effect, landscape configuration shapes resource distribution, rendering certain zones much more prone to predation than others. In terrestrial ecosystems, for example, many woodland bird assemblages can suffer major declines as forest areas decrease and fragment, exposing birds to predator incursion [48,49]. Similarly, dense patchy fragments are potentially easier places for fish predators to wander between seagrass patches, picking off the urchins within them [50,51]. *P*. *oceanica* fragmented meadows can potentially influence the first step of top-down control by modifying the distribution of predation risk on herbivore sea urchins in relation to patch aggregation. Distribution of predation risk by fish seems to reflect the aggregation in space of high perimeter-to-area ratio patches (predation hotspots), according to the state of fragmentation of the habitat [52].

Seagrasses comprise some of the most heterogeneous shallow-water ecosystem landscapes in the world where our understanding of animal responses to variability in seagrass landscape structure is still fragmentary [53]. This study tested whether the predation risk of juvenile *P*. *lividus* (measuring survival of tethered animals) changes in relation to different levels of *P*. *oceanica* fragmentation and the spatial arrangement of seagrass patches. This aim is consistent with the goals of landscape ecology, which investigates the ecological consequences of broad-scale spatial heterogeneity and the dynamics of biotic and abiotic processes over large areas. In the shallow subtidal zone, a mosaic of seagrass habitats (habitat classes) can be found: ‘continuous’ (no fragmentation) seagrass habitat, ‘low’ and ‘high’ fragmentation and ‘rocks’ with no seagrass. In this study the expectations were that juvenile *P*. *lividus* survival would be higher i) in the continuous *P*. *oceanica* habitat due to the low fish predation pressure and ii), at the landscape level, in the area with the highest occupancy of continuous *P*. *oceanica* (least fragmentation). To this aim, the spatial configuration of habitat patches was defined by elaborating a detailed map of the seabed using both Geographic Information System (GIS) and field surveys. These approaches allowed for the merging of habitat structural information into the landscape scale.

## Materials and Methods

### Study area and mapping

**Fig 1:**
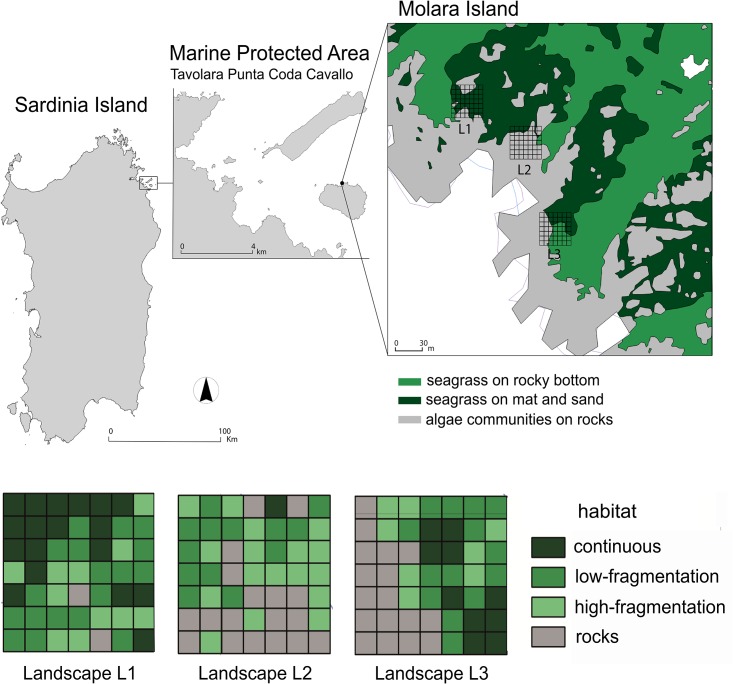
Map of the Island of Sardinia showing the Marine Protected Area and corresponding study site. Tavolara Punta Coda Cavallo Marine Protected Area (Sardinia Island; 40°52’22” N, 9°44’27” E). Three landscapes are mapped (L1, L2 and L3), four habitat classes represent the levels of seagrass fragmentation in each cell. Reprinted from the biocenosis map of the archives of the MPA under a CC BY license, with permission from the managing Director of Tavolara Capo Coda Cavallo AMP, original copyright 2015.

The experiment was conducted in the MPA of Tavolara Punta Coda Cavallo (Sardinia, Western Mediterranean; 40°52’22” N, 9°44’27” E) with the permission of the management board. The study site is within the no-take zone (North side of Molara Island; Fig 1) and is characterized by both oligotrophic conditions and high density fish assemblages that have been restructured in 15 years of effective protection [54] (Fig 1).

At this site (9 m deep on average) a mosaic of patches of different levels of *P*. *oceanica* fragmentation can be found. *P*. *lividus* adult density could only be quantified on rocky bottoms (2.0 ± 0.34, mean ± SE individuals m^-2^), while it was extremely infrequent in *P*. *oceanica* habitats. Recruitment was not estimated at the study site. Nevertheless, during an extensive sampling effort conducted recently, an overall paucity of recruits (individuals < 20 mm) was found in the reserve area of this MPA, recruits being aggregated in a very small number of quadrats [55]. This feature is absolutely consistent to other oligrotrophic and protected areas where low recruitment is determined by low larval supply [36] and high fish predation for the reserve effect [56].

Furthermore, the abundance of benthic predators was evaluated by placing cages with a piece of raw beef on the sea bottom (this being the most common whelk fishing technique in the area) in different areas of the site: 3 ± 0.6 and 4 ± 0.3 (mean ± SE, n = 3) *Hexaplex trunculus* (Linnaeus, 1758) individuals were found in those cages placed at continuous and patchy meadows respectively, while no whelks were found on cages positioned on the rocks (S2 Fig).

With the aim of relating sea urchin predation risk to different levels of seagrass fragmentation (habitat class) and their spatial arrangement, three square areas (35×35 m^2^) about 100m apart were identified as different landscapes (L1, L2 and L3, Fig 1). Each square area was divided into a grid of 49 cells (5×5 m^2^ each) whose GPS-coordinates were pinned up on a digital map with Free and Open Source Geographic Information System (QGIS) and, successively, identified on the seafloor and marked with previously labelled bricks. The cell size was the finest spatial resolution we could consider to detect differences in relation to seagrass fragmentation (S1 Fig).

Underwater inspection of seagrass presence was conducted in every cell, so that each cell could be assigned to a level of seagrass fragmentation as a different habitat class. A gradient of four habitat classes was defined: 1) continuous, 2) low-fragmentation (few large patches), 3) high-fragmentation (many small patches) and 4) rocks (absence of *P*. *oceanica*). With QGIS, the adjacent cells with the same level of habitat fragmentation were joined to produce a layer map of patches belonging to the same habitat class. Finally, the patches differed in shape and size and, consequently, the minimum mapping unit (*i*.*e*., grain size) corresponded to the minimum patch size of the habitat class that was mapped.

All habitat classes were present in each area, although their importance and arrangement differed considerably (Table 1 and Fig 1). Specifically, L1 was mainly composed of ‘continuous’ and ‘low-fragmentation’ habitats, L2 mainly by ‘high-fragmentation’ and ‘rocks’ habitats, while in L3 the presence of the four habitat classes was more balanced. Therefore, L1, L2 and L3 corresponded to three different levels of landscape heterogeneity. The size of each square area was determined as the best compromise between the heterogeneity of the habitat class and the number of cells necessary for spatial analysis [57].

**Table 1:** Landscape attributes for each level of fragmentation.

	Habitat class	NP	PD(100 m^-2^)	MPA(m^2^)	GPA(m^2^)	PC(%)	MPC(m^-1^)	IJI(%)
L1	continuous	3	0.24	150	375	36.7	0.6	59.5
	low-fragmentation	2	0.16	225	425	36.7	0.6	84.6
	high- fragmentation	5	0.4	55	125	22.5	0.7	88.9
	rocks	2	0.16	25	25	4.1	0.8	62.2
L2	continuous	1	0.08	25	25	2	0.8	57.9
	low-fragmentation	2	0.16	163	225	26.5	0.5	68.5
	high- fragmentation	6	0.5	71	300	34.7	0.7	63.1
	rocks	3	0.24	150	400	36.7	0.7	74.3
L3	continuous	2	0.16	125	150	20	0.4	40.4
	low-fragmentation	1	0.08	350	350	28.6	0.6	89.3
	high- fragmentation	3	0.24	67	75	16.3	0.6	73.6
	rocks	1	0.08	425	425	34.7	0.3	80.8

Attributes describing spatial arrangement of the four habitat classes in the landscapes L1, L2, L3. (NP) Number of Patches; (PD) Patch Density; (MPA) Mean Patch Area; (GPA) Greatest Patch Area; (PC) Patch Cover; (MPC) Mean Patch Complexity and (IJI) Interspersion/Juxtaposition Index.

### Landscape attributes

The spatial pattern of each habitat class was described using spatial indices containing information relevant to the evaluation of landscape fragmentation. They were computed with Fragstats 4.1 on the basis of the patch information. In general, spatial indices may describe patch composition, shape, or configuration [58]. Here the Number of Patches of *P*. *oceanica* (NP), Patch Density (PD, in 100 m^2^), Mean Patch Area (MPA, m^2^), Greatest Patch Area (GPA, m^2^), Patch Cover (PC, %), Mean Patch Complexity (MPC, 1/m), and Interspersion/Juxtaposition Index (IJI, %) of habitat classes were calculated (Table 1). Among these, PD, MPC, and IJI were selected as descriptors of composition, shape, and configuration of the habitat classes, respectively. PD determines the basic characteristics of fragmentation because it describes the number of patches for each habitat class in a specific area (100 m^2^) and includes information regarding both the number of patches and patch areas. In order to quantify patch complexity (*i*.*e*., perimeter-to-area ratio), the MPC of each habitat class was calculated as the mean of the shape complexity of their patches, with high values indicating higher complexity. Finally, IJI was selected to describe patch interspersion over the maximum possible interspersion for the given number of patches of different seagrass habitat classes [59]. High values result from landscapes in which patches of a habitat class are well interspersed, whereas lower values characterize landscapes in which patches of a habitat class are poorly interspersed. IJI would be 100 where patches of a habitat class are equally adjacent to all other patch types (maximum interspersion).

### Structural attributes

**Fig 2:**
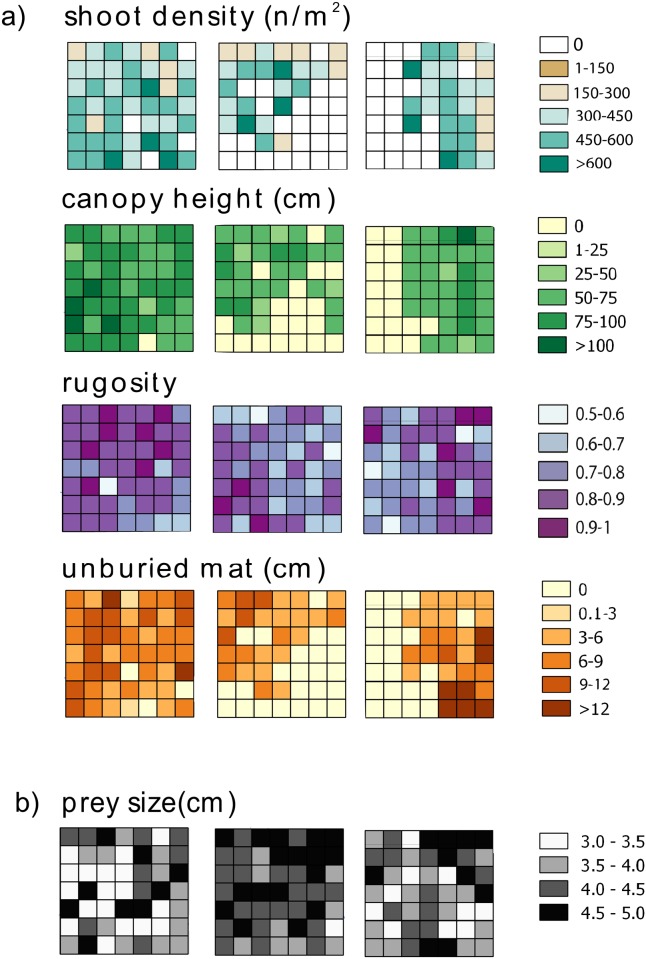
Spatial distribution of predictor variables. Spatial distribution of predictor variables within each landscape (L1, L2 and L3): structural attributes (shoot density, canopy height, rugosity and unburied mat,) and prey size.

To describe *P*. *oceanica* structure in each cell, several structural attributes were measured ad hoc in the field (Fig 2). Shoot density, canopy height, and height of unburied mat were measured [60]. Shoot density was estimated on the basis of a 50×50 cm^2^ quadrat randomly placed; seagrass canopy height was measured as the longest leaf in randomly selected shoots (n = 3); height of unburied mat was measured as the distance between the leaf base (ligula) and the sediment surface (n = 3). Moreover, substrate complexity (hereafter ‘rugosity’) was calculated by means of the rope-and-chain method [61]: rugosity corresponds to the difference between the length (5 m) of a stainless-steel chain and the measured distance between the two ends after placing it on the bottom and letting it adapt to the irregularities of the substrate. Each value was obtained by dividing the distance between the two ends by the length of the chain, in order to scale values between 0 and 1, where values close to 0 indicate higher rugosity and higher values correspond to less rugose and smoother substrates.

### Predation risk

Predation risk was estimated during late summer when fish predator activity is at its maximum [56]. Sea urchins of 3 to 5 cm (test diameter, TD) were collected in rocky habitats near the study site, measured, and then tethered using the piercing technique [62]. Each sea urchin was randomly assigned to one cell (Fig 2), then hooked with a 50 cm-long fishing line to a labelled brick placed in the center of each cell, and left fairly free to move around the brick to look for shelter within a 50 cm radius area [62,63]. The effects of prey manipulation associated with this tethering technique under the conditions of the experiment was negligible, as only 8.1% of prey died within a few hours (loss of all spines) due to stress (12.2% in L1, 4% in L2, 8.1% in L3; see results).

Sea urchin survival was checked daily. The experiment ended when prey predation reached 60% in at least one area. Survival rate for each individual was estimated as the ratio between the number of days survived and the duration in days of the experiment. The predation rate at the end of the experiment was calculated as 1 − survival rate (expressed in a scale ranging from 0 to 1) and represented in layer maps. The type of attack was also classified depending on the type of mark found: fishing line loop without sea urchin or broken skeleton was classified as fish attack, while a drill hole found on prey skeleton indicates benthic predator attack [44].

### Survival analysis

The analysis of survival was based on the time taken for predation to occur [64,65]. Once time-to-event data were collected, the survival among landscapes and among habitat classes within each landscape was compared: the LogRank-test was used to compare survival curves, while Cox proportional-hazards regression model (Coxph-test) was used to identify alternative hypotheses [64].

Coxph-test [66] is the most widely used method of survival analysis to examine the relationship between survival and one or more predictors on failure time. Results of this analysis were used to evaluate the risk of predation and the contribution of each covariate. First, a Full Coxph Model considering all structural attributes (shoot density, canopy height, height of the unburied mat and rugosity), plus prey size and landscape attributes (PD, MPC, IJI) as predictors was used. Redundant covariates were excluded by means of Spearman tests. Then a Minimal Adequate Model with the minimal number of covariates influencing predation risk was obtained through a stepwise forward regression procedure. The goodness-of-fit of the model was compared with the earlier versions using Akaike’s Information Criterion (AIC) and likelihood ratio tests [67]. All analyses were performed using Survival Analysis package for R software (R Development Core Team 2010) [65, 68].

## Results

**Fig 3:**
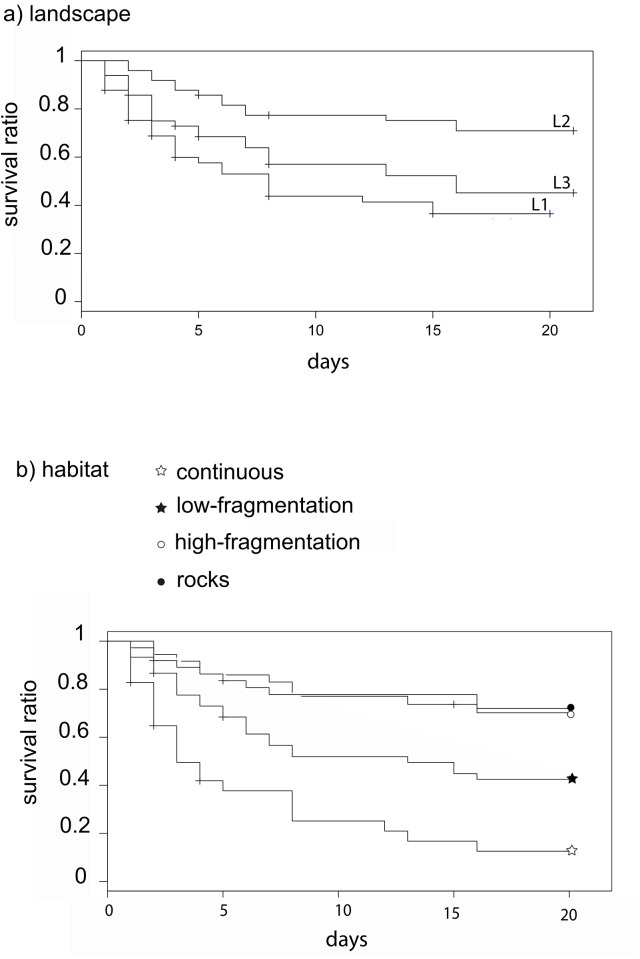
Survival curves among landscape areas and habitat classes. Significant differences were observed for urchin survival among (a) landscape areas (L1, L2 and L3) and (b) habitat classes over 20 days. Levels of significance are represented in Table 2.

**Fig 4:**
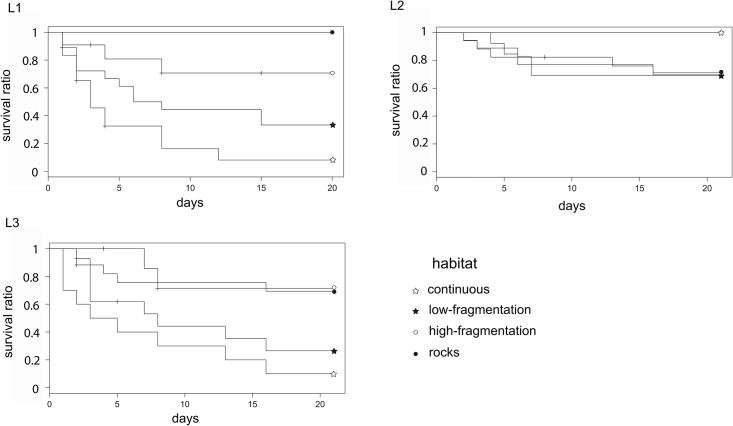
Survival curves among habitat classes within each landscape area. Significant differences were observed for urchin survival among habitat classes within each landscape area during 20 days. Levels of significance are represented in Table 3.

Twenty days after sea urchin positioning, predation rate was 67.5%, 30% and 57% in L1, L2 and L3 landscape areas, respectively. Overall, a landscape effect was detected as sea urchin survival was significantly higher in L2, the area where the ‘rocks’ habitat had the highest presence, rather than in L1 and L3 (Fig 3a and Table 2). The predation risk was also affected by the habitat class: survival was significantly lower in the ‘continuous’ seagrass (11.5%) than in all other classes (42% ‘low-fragmentation’, 68% ‘high-fragmentation’ and 71% on rocks; see Fig 3b and Table 2). In L1 and L3 survival was significantly lower both in the ‘continuous’ and ‘low-fragmentation’ habitats, while in L2 there was no difference among habitat classes and survival on ‘rocks’ was as high as in the ‘continuous’ seagrass (Table 3 and Fig 4).

**Table 2:** Coxph-test for predation differences among landscapes and habitat classes.

Response variable	model	effect	coefficient	z-value	p-value
Predation risk	~ landscape + habitat	L2	-0.67243	-1.939	0.05252
		L3	-0.07917	-10.281	0.778854
		low-fragmentation	-0.72372	-2.434	0.014923 *
		high-fragmentation	-1.54057	-3.920	8.87e-05 ***
		rocks	-1.57249	-3.887	0.000102 ***

Level of significance is represented with the asterisks “*”, “**”, “***” (R^2^ = 0.22; Log-rank test p = 4.007e).

**Table 3:** Coxph-test for predation differences among habitat classes within each landscape area.

Landscape area	Response variable	model	effect	coefficient	z-value	p-value
L1	Predation risk	~ habitat class	low-fragmentation	-7.033e-01	-1.728	0.08408
			high-fragmentation	-1.791e+00	-2.767	0.00566 **
				-1.902e+01	-0.003	0.99764
L2	Predation risk	~ habitat class	rocks	1.708e+01	0.002	0.998
			high-fragmentation	1.705e+01	0.002	0.998
			rocks	1.700e+01	0.002	0.998
L3	Predation risk	~ habitat class	low-fragmentation	-0.5821	-1.233	0.21760
			high-fragmentation	-1.8780	-2.397	0.01653 *
			rocks	-1.7220	-3.055	0.00225 **

Level of significance is represented with the asterisks “*”, “**”, “***” (L1: R^2^ = 0.25; Log-rank test p = 0.005; L2: R^2^ = 0.014; Log-rank test p = 0.947; L3: R^2^ = 0.024; Log-rank test p = 0.019).

The high percentage of prey surviving during the whole experiment determined a limited predictive power of the model (Coxph-test), which explains roughly 30% of the variance (Table 4). Among all the covariates considered, MPC was the only one which significantly influenced predation risk (i.e., the lower the area, the higher the survival, AIC = 114.6; Table 4). Thus, none of the structural attributes of the habitat and neither prey size affected the survival of sea urchins.

**Table 4:** Coxph-test used to evaluate the contribution of the covariates (structural attributes, landscape attributes and prey size) to the sea urchin predation risk distribution.

Response variable	Full model	Selected model	coefficient	z-value	p-value
Predation risk	~ size + density + canopy + rugosity+ mat + MPC + IJI + PD	MPC	-7.907548	-2.323	0.0202 *

The Minimal Adequate Model (AIC = 114.6) was obtained starting from Full Model (AIC = 118.6) through the stepwise forward regression technique. Level of significance is represented with the asterisks “*”, “**”, “***” (R^2^ = 0.28; Log-rank test p = 0.011).

**Fig 5:**
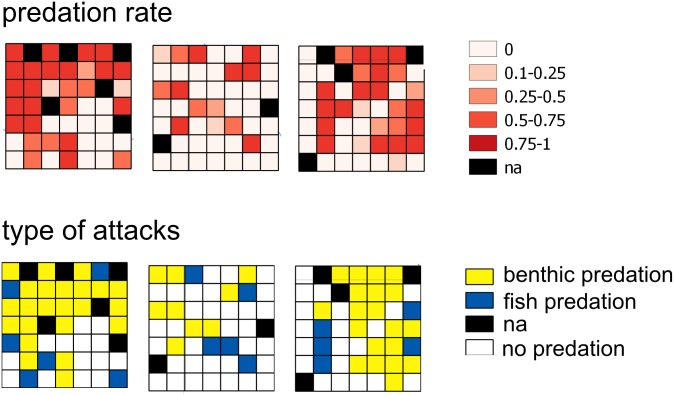
Spatial distributions of final predation rate and type of predator attacks. Spatial distribution within each landscape area (L1, L2 and L3) of the final predation rate and type of predator attack suffered by sea urchins. Also, “na” means no available data (dead prey due to stress, see M&M).

At the end of the experiment, fish predation on ‘rocks’ only contributed 8.8% of the overall attacks on sea urchins. The highest predation activity was estimated in the ‘continuous’ and ‘low-fragmentation’ *P*. *oceanica* (Table 1). In these habitats, mark types found on the sea urchins suggested that the most frequent type of attack (79% and 90% in ‘continuous’ and ‘low-fragmentation’, respectively) was due to benthic predators attributable to whelks (*Muricidae* spp., Fig 5). Conversely, this type of attack was extremely rare in the ‘rocks’ habitat. Overall, 76.5% of the pooled attacks on juvenile sea urchins were due to benthic predators, and this outcome matches whelk field distribution estimated by fishing cages (S2 Fig).

## Discussion

Predation risk for juvenile sea urchins differed across landscapes, being significantly lower in areas with the higher presence of ‘rocks’. This is also in accordance with the effect of habitat classes on predation that clearly indicated a higher risk in the ‘continuous’ *P*. *oceanica* rather than in the other classes. Therefore, the expectation that juvenile *P*. *lividus* survival would have been higher in the ‘continuous’ seagrass habitat was not met. Predation risk also changed across habitats in terms of attack types: as benthic predator attacks were, overall, associated with the ‘continuous’ seagrass habitat. Finally, mean *P*. *oceanica* patch complexity (one of the landscape attributes) had a negative effect on sea urchin predation. Accordingly, the low predation activity in the ‘continuous’ habitat of L2 was probably due to the absence of large seagrass patches.

Predation in seagrass was mostly due to whelks: more than 70% of predated sea urchins were observed with a regular drilled hole in their skeleton. Several studies indicate that whelks can feed on a range of species of barnacles, bivalves, gastropods, tunicates, bryozoans [44], and sea urchins [56,69]. They typically access their prey either by drilling the shell or by marginal chipping, aided by acid secretion [44]. Surprisingly, in this study their activity was associated with ‘continuous’ *P*. *oceanica*. Because *H*. *trunculus* was the only whelk found in our surveys, we believe it is the species most probably responsible for the benthic attacks on the tethered sea urchins. This whelk has been observed to occur both inside *P*. *oceanica* beds and on rocky bottoms [44]. However, at the study site it was tightly confined inside the seagrass, whose edges might not be a barrier for this generalist predator/scavenger. Thus, behavioural observation of species–habitat relationships highlights the need to investigate the spatial relationship between the habitat and the dynamics of *H*. *trunculus* population. We suspect that the high concentration of this gastropod in the ‘continuous’ habitat could be due to the lack of shelters from eventual predators successfully acting on the rocks. Effectively, the very well-structured community of fish predators, as a consequence of the strong protection effectiveness of the MPA, may force the gastropod to select the seagrass, either to avoid predation in the open space and/or to search for feeding resources [54].

Seagrass structure can operate both as safe shelter from visual fish predators and as feeding ground for benthic predators that use it for ambush and camouflage [14,15]. When benthic predators are abundant, they are generally proportional to the dimensions of the patch size. Similar to the results of this study, Hovel and Lipcius (2001) found that patch size drastically decreases the survival of blue crabs due to a greater abundance of benthic predators in ‘continuous’ habitat [70]. Thus, in order to identify the distribution of predation hotspots across landscapes, it is of paramount importance to estimate the local occurrence of predators of different guilds [17].

Overall, sea urchin survival estimates between habitats were positively related to the adult population density (*i*.*e*., low abundance on rocks vs. unappreciable abundance in *P*. *oceanica*), thus identifying top-down forces as the determinants to such population distribution. In other words, survival estimates undoubtedly demonstrate the importance of predation (distinguishing between the two types) on the *P*. *lividus* adult distribution, discouraging any future investigation on the population recruitment limitation at this site. In fact, both the oligotrophic conditions and the high fish predator density could indirectly contribute to a general low sea urchin population density, by lowering larval supply [36] and reducing settler abundance [55], respectively. Also, higher *P*. *lividus* settlement is predicted on the rocky shallower bottom than in seagrass meadows [42] and recruitment usually limits populations in seagrass meadows [55].

However, at this site we believe that the sea urchin abundance and distribution between habitats could be the result of the seagrass effect on their predation risk, which would be turned upside down. In fact, in a mosaic of shallow subtidal rocky/seagrass habitats, high density large-sized *P*. *lividus* are frequently found in *P*. *oceanica* edges, as they move from rocks towards the seagrass in search of shelter rather than resources [37,39]. It is well known that the perception of high fish predator density induces sea urchins to migrate deeper towards the seagrass edges, where there is the possibility to benefit from the shelter offered by a structured seagrass canopy [41]. Therefore, the risk effect of one predator would eventually shift the prey into a ‘safer’ habitat, where the predation risk from other predators should decrease. However, this concept may not be true in multi-predator systems, especially if there are predators with different strategies. For example, in large carnivore-ungulate interactions, where there are both stalking and cursorial predators, the risk effect of one predator should increase the predation risk from another predator inducing a prey habitat shift [71]. Effectively, the capacity to perceive predators has strong behavioural consequences on prey, which in turn modifies other interactions and, ultimately, the abundance, distribution and interactions with the environment of a species [11]. However, whether the ‘landscape of fear’ concept, in which prey animals are aware of varying levels of predation risk at a given spatial scale, has not thus far been explored for sea urchins. The ‘landscape of fear’ would commonly be highlighted by a negative spatial relationship between prey and predator, in which prey purposely avoid the riskiest sites in the landscape. Yet, the lack of sea urchins in the ‘continuous’ seagrass could be the result of either an active avoidance of the habitat or the effect of a pressing predation exerted on the prey by whelks.

Furthermore, there is also a different effect that the two predator guilds could produce on *P*. *lividus* population structure. Although fish predation can greatly affect small size individuals in strong relation with their own size [72], there is little evidence that whelks are size-selective predators. In this study prey-size did not significantly affect sea urchin survival, probably due to the higher proportion of attacks by benthic predators, which have no reservations about drilling even the largest sea urchins. Accordingly, all predators control sea urchin population abundance, but, only fishes would affect the population structure of *P*. *lividus*, while benthic predators would make any size individuals vulnerable, potentially triggering dramatic declines in the sea urchin population. Thus, there are sites within this MPA (Tavolara Punta Coda Cavallo) where benthic predators play a pivotal role in directly shaping sea urchin population abundance, and where macrophyte communities resemble more a ‘death trap’ than a protective habitat [73].

In conclusion, fish predation on juvenile sea urchins on ‘rocks’ and ‘high-fragmentation’ seagrass habitats was less likely than benthic predation in the ‘continuous’ seagrass, with low seagrass patch complexity increasing benthic activity. Therefore, at the landscape level, seagrass fragmentation negatively influenced benthic predation on sea urchins. Since fish predator activity on urchins has been shown to increase with fragmentation [52], the effect of patch complexity can be controversial depending on the local composition of predator guilds. Future research should be aimed at investigating if, derived from the complex indirect interactions among species, top-down control in Marine Reserves can modify seagrass habitat effects.

## Supporting Information

S1 Fig Landscape square example. Each square (35 × 35 m^2^ landscape area) was divided in 49 cells. The sampling unit corresponds to a cell of 5 × 5 m^2^. Contiguous group of cells of the same class of habitat represents a patch.
(TIFF)

S2 Fig Benthic predators’ distribution. Average abundance (n = 3) of *Hexaplex trunculus* individuals found in the fishing cages placed in the continuous meadow, patchy meadow and rocks.
(TIFF)

S1 File Work authorization and copyright. (PDF)
