# Supplementary figures and images for: The Seagrass Effect Turned Upside Down Changes the Prospective of Sea Urchin Survival and Landscape Implications
Source: PLoS One. 2016 Oct 26;11(10):e0164294. doi: 10.1371/journal.pone.0164294 (PMC5082627; doi:10.1371/journal.pone.0164294)

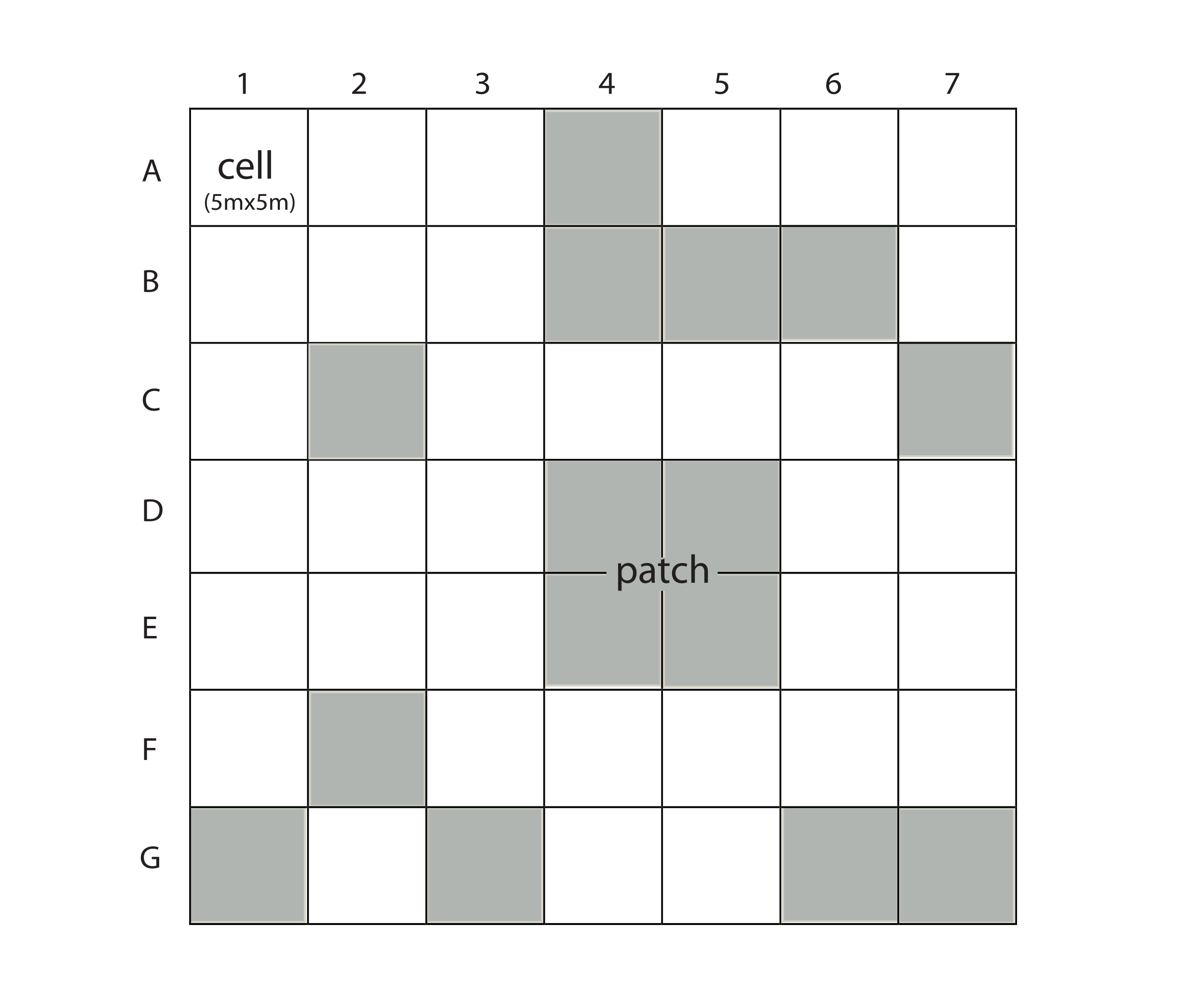

Supplement: S1 Fig — Each square (35 × 35 m2 landscape area) was divided in 49 cells. The sampling unit corresponds to a cell of 5 × 5 m2. Contiguous group of cells of the same class of habitat represents a patch. (TIFF) [file pone.0164294.s001.tiff]

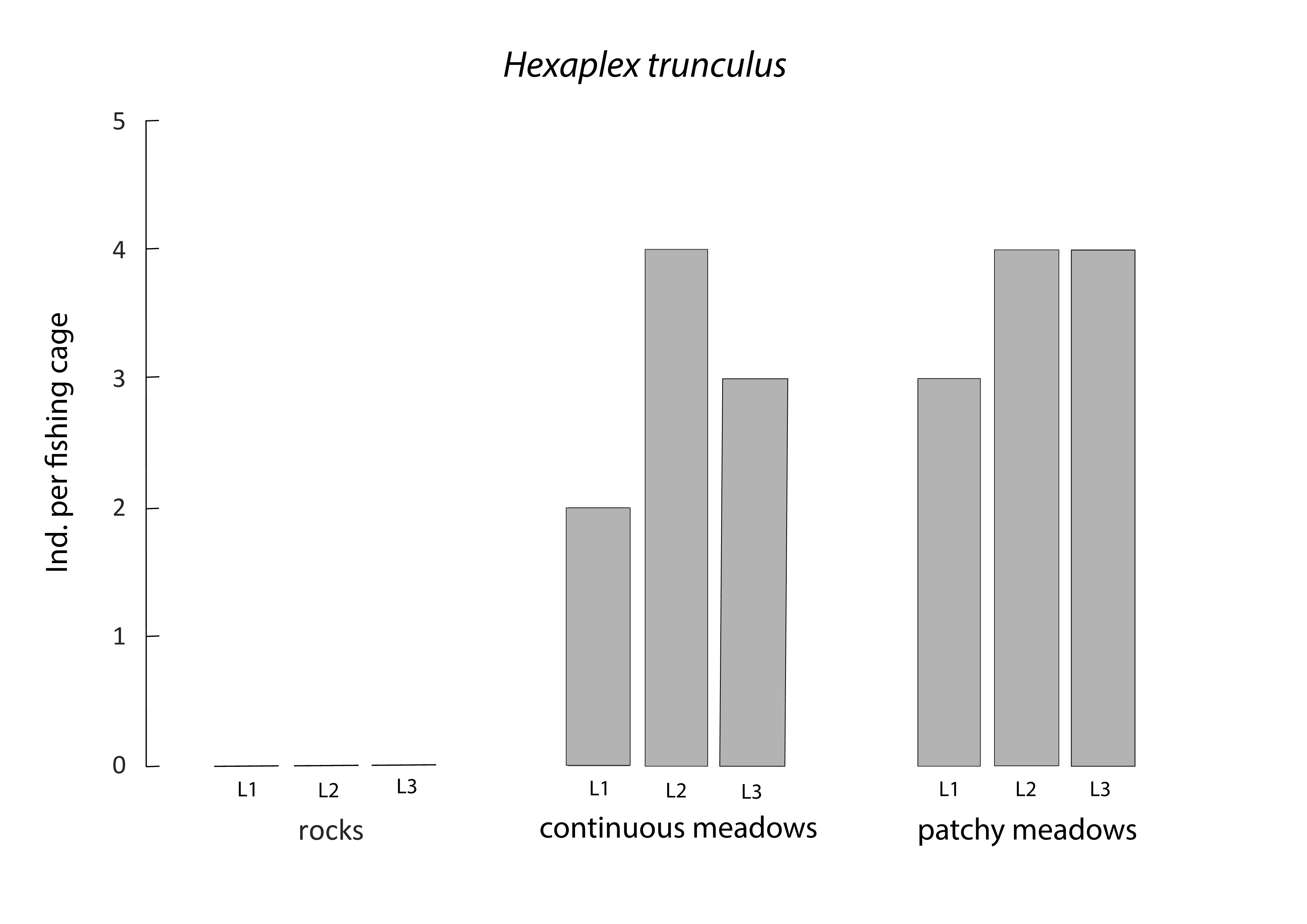

Supplement: S2 Fig — Average abundance (n = 3) of Hexaplex trunculus individuals found in the fishing cages placed in the continuous meadow, patchy meadow and rocks. (TIFF) [file pone.0164294.s002.tiff]
