# Supplementary material for: The Seagrass Effect Turned Upside Down Changes the Prospective of Sea Urchin Survival and Landscape Implications
Source: PLoS One. 2016 Oct 26;11(10):e0164294. doi: 10.1371/journal.pone.0164294 (PMC5082627; doi:10.1371/journal.pone.0164294)
Supplement: S1 File — (PDF) [file pone.0164294.s003.pdf]

AREA MARINA PROTETTA
TAVOLARA
PUNTA CODA CAVALLO

MINISTERO DELL'AMBIENTE

Consorzio di Gestione Area Marina Protetta di Tavolara Punta Coda Cavallo

AUTORIZZAZIONE 2014

Il Direttore dell'Area Marina Protetta di "Tavolara – Punta Coda Cavallo", Dott. Augusto Navone, Responsabile della gestione della medesima,

VISTA La richiesta di autorizzazione presentata dalla dr.ssa Giulia Ceccherelli, in qualità di ricercatrice del Dipartimento di Scienze della Natura del Territorio dell'Università di Sassari, e responsabile del progetto Regionale "Promozione della ricerca scientifica e dell'innovazione tecnologica in Sardegna", "Approccio integrato per la tutela, la gestione e la valorizzazione della risorsa riccio di mare in Sardegna";

VISTA la Delibera del C.d.A. n.9 del 24 Febbraio 2006, che adottava i criteri transitori per l'erogazione di pareri ambientale sul demanio costiero;

VISTO il verbale della Commissione di Riserva del 6 Marzo 2006 che ratificava l'adozione dei cateteri transitori sopra richiamati;

VISTO l'art. 1 dell'intesa in data 14/09/2005 fra Regione, Ministero dei Trasporti e Infrastrutture e Ministero dell'Ambiente in ordine al rilascio o rinnovo di titoli concessori all'interno dell'AMP;

VISTA la delibera dell'assemblea consortile n. 9 del 21/12/06 , con cui si approva il Piano di Gestione dell'AMP;

VISTA l'ordinanza n°21/2014 della Capitaneria di Porto di Olbia;

VISTA la L. 31.12.1982 n. 979;

VISTA la L. 6.12.1991 n. 394;

VISTA la Direttiva 92/43/CEE del consiglio del 21 maggio 2992 relativa alla conservazione degli habitat naturali e seminaturali e della flora e della fauna selvatiche e ss.mm.ii;

VISTO il D.P.R. n. 357/1997, art. 3, comma 1, e successive modifiche ed integrazioni individuazione di nuovi siti Natura 2000 a mare;

VISTA la Deliberazione della Regione Autonoma della Sardegna n. 21/62 del 03/06/2010, che identifica il SIC Isola di Tavolara, Molara e Molarotto ITB 010010 coincidente con il perimetro dell'AMP;

VISTA la Convenzione per l'affidamento di alcune funzioni relative alla gestione dei siti della rete natura 2000 Rep. N° 12459-81 A.D.A del 28/05/2012.

AUTORIZZA

La dr.ssa Giulia Ceccherelli, e la sua equipe, ad effettuare attività di ricerca e prelievi di *Paracentrotus lividus* in tutto il territorio dell'AMP di Tavolara Punta Coda Cavallo, Escluse le zone A. La presente autorizzazione ha validità dal 01 Agosto 2014 al 30 Settembre 2014. L'imbarcazione utilizzata per la ricerca sarà il natante tipo GOMMONAUTICA G 48, di l.f.t. 4.80 m.. Con un motore Yamaha da 40 Cavalli mat. 6H4-304287.

Tutte le operazioni dovranno essere svolte in rispetto delle vigenti norme previste dall'AMP

CONSORZIO DI GESTIONE A.M.P. DI TAVOLARA
PUNTA CODA CAVALLO
PROTOCOLLO GENERALE
31/07/2014
N° 0001246 [illegible]

A.M.P. TAVOLARA CONSORZIO DI GESTIONE PUNTA CODA CAVALLO

Il Direttore
Dr. Augusto Navone

Marine Protected Area Of Tavolara Punta Coda Cavallo

As Director of Tavolara Capo-Coda Cavallo Marine Protected Area (North Sardinia, Italy) in charge of the same management

And after the request of permission of Simone Farina, author of the manuscript entitled, " The Seagrass effect turned upside down changes the prospective of sea urchin survival and landscape implications" to use a portion (approximatively 200m^2) of the biocenosis map of Molara Island coming from the archives of the MPA and realized during the year **2015**

I, the undersigned, hereby authorize the open-access journal PLOS ONE to publish the biocenosis map represented in Fig.1 under the Creative Commons Attribution License (CCAL) CC BY 4.0

The director of MPA
Dott. Augusto Navone

A.M.P. TAVOLARA - CONSORZIO DI GESTIONE - PUNTA CODA CAVALLO

Olbia , 05/08/ 2016
